# A Prospective cohort study of zoonotic cutaneous leishmaniasis in tunisia: Clinical and Immunological features and immune correlates of protection

**DOI:** 10.1371/journal.pntd.0011784

**Published:** 2023-12-08

**Authors:** Ikbel Naouar, Wafa Kammoun Rebai, Afif Ben Salah, Hind Bouguerra, Amine Toumi, Nabil Belhadj Hamida, Hechmi Louzir, Amel Meddeb-Garnaoui

**Affiliations:** 1 Laboratory of Transmission Control and Immunobiology of Infection, Pasteur Institute of Tunis, Tunis, Tunisia; 2 Faculty of Sciences, University of Tunis El Manar, Tunis, Tunisia; 3 Department of Immunology, University of Toronto, Ontario, Canada; 4 Laboratory of Medical Parasitology, Biotechnology and Biomolecular, Pasteur Institute of Tunis, Tunis, Tunisia; 5 Department of Family and Community Medicine, College of Medicine and Medical Sciences, Arabian Gulf University, Manama, Bahrain; 6 National Observatory of New and Emerging Diseases, Tunis, Tunisia; 7 Faculty of Medicine of Tunis, University Tunis El Manar, Tunis, Tunisia; 8 Health Information and Intelligence Section, Ministry of Public Health, Doha, Qatar; Hebrew University-Hadassah Medical School, ISRAEL

## Abstract

**Background:**

This study aimed to define immunological markers of exposure to *L*. *major* parasites and identify correlates of protection against infection.

**Methods:**

We analyzed a cohort of 790 individuals at risk of developing ZCL living in endemic areas with varying *L*. *major* infection prevalence. One area had a high infection prevalence indicated by high proportions of leishmanin skin test (LST) positive subjects, while the other areas were recent foci with lower infection prevalence. Blood samples were collected before the transmission season to measure Interferon gamma (IFN-γ), Interleukin 10 (IL-10), and Granzyme B (GrB) levels in response to parasite stimulation in peripheral blood mononuclear cells.

A one-year follow-up period involved active detection of new ZCL cases to estimate disease incidence after a transmission season and identify immune correlates of protection.

**Results:**

The study population showed heterogeneity in parasite contact, evident from specific scars and/or positive LST results, significantly higher in the old focus compared to recent foci. IFN-γ and GrB were markers of parasite exposure and reliable indicators of immunity to *L*. *major*. Positive correlations were observed between IFN-γ/IL-10 and GrB/IL-10 ratios and LST results.

Unexpectedly, only 29 new ZCL cases (4%) appeared after a transmission season, with 27 cases reported in recent foci and 2 in the oldest focus. Our findings indicate that individuals in *L*. *major* endemic areas are likely to develop ZCL regardless of their LST status.

We showed that high pre-transmission season levels of IFN-γ and GrB produced by PBMC, along with a high IFN-γ/IL-10 ratio, were associated with protection.

**Conclusion:**

This study on a large cohort at risk of ZCL confirmed IFN-γ and GrB as protective factors against the disease. A high IFN-γ/IL-10 ratio, but not GrB/IL-10 ratio was associated with resistance. These results are valuable for developing and evaluating of a vaccine against human leishmaniasis.

## Author summary

A cohort of 790 individuals at risk of developing ZCL, residing in areas endemic to leishmaniasis with different prevalence of *L*. *major* infection, was followed for a one-year period to determine the incidence of the disease following a transmission season and to identify immune correlates of protection.

Our results showed that the study population in an endemic area exhibited heterogeneity in terms of exposure to the bites of infectious flies due to the characteristics of the houses such as the presence of shelters for animals which attracts the vectors. IFN-γ and GrB were detected in most participants with significantly higher values in the old focus compared to recent foci and among individuals with LST reaction. Furthermore, a strong correlation was observed between IFN-γ and GrB as well as a positive association between the IFN-γ/IL-10 ratios and LST results. Cumulative incidence of infection among the study cohort was 4% after a transmission season, primarily from recent foci. We also showed that high levels of IFN-γ and GrB produced by PBMCs in response to the parasite, as well as a high IFN-γ/IL-10 ratio, at the steady state, were associated with protection against disease development.

## Introduction

Leishmaniasis is recognized as one of the top ten neglected diseases worldwide, underscoring a critical global public health challenge [1]. The cutaneous form of this disease is also a major public health problem worldwide. Zoonotic cutaneous leishmaniasis (ZCL), caused by *Leishmania (L*.*) major*, is endemic in Tunisia, where the epidemic emerged since 1982 in central Tunisia and expanded to many parts of the country, with the emergence of several new foci and constitutes a public health problem [2, 3]. A total of 2219 new ZCL cases/year have been recorded between 2013 and 2015 according to the statistics of the national program of cutaneous leishmaniasis control of the Ministry of Health. The parasite *L*. *major* is transmitted by female sand fly vectors, *Phlebotomus papatasi*, with a transmission season during the summer months (May to September). In fact, there are two peaks of density of sandflies in June and September, however most of flies are found infected with the *Leishmania* parasites in the latter. Infected humans develop the full-blown disease as lesions in exposed parts of the body with an incidence peak from October to December [4].

ZCL is a polymorphic disease with various clinical manifestations ranging from asymptomatic infection to benign self-healing or more extensive and disfiguring lesions [5–7]. The common concept of the natural history of *L*. *major* infection assumes that an infecting sand fly bite often results in the development of cutaneous lesions leaving scars after healing. The recovery from leishmaniasis is based on a cell-mediated immune response which can be evaluated by a parasite specific delayed-type hypersensitivity (DTH) reaction. This response is typically observed by a positive leishmanin skin test (LST) reaction. Most individuals living in areas endemic for cutaneous leishmaniasis (CL) due to *L*. *major*, who recover from a primary infection, develop a lifelong immunity to reinfection, although cases of reinfection have been documented.

The *L*. *major* murine model has provided an operational definition of protective cell-mediated immunity against *Leishmania* parasites. It has established the crucial role of CD4+ T cells Th1, associated with interferon-γ (IFN-γ) and Tumor Necrosis Factor (TNF-α) production, in resistance and Th2, associated with Interleukin 4 (IL-4), IL-5, IL-13, IL-10 and Transforming growth factor β (TGF-β), in susceptibility to *Leishmania* infection [8–11].

It should be noted, however, that while Th1 cells are crucial for protection, they may not be sufficient [12].

In humans, the characterization of the immuno-pathological and immuno-protective mechanisms during infection is challenging because of the heterogeneity of the genetics in human and in the parasite populations. However, the prevailing view is that Th1 responses are essential for the control of parasite replication and are crucial for vaccine development.

Th1 CD4+ T cells producing IFN-γ and positive DTH responses have been associated with healing of CL [12–16]. CD8+ T cells are also important for healing mainly through IFN-γ production [17–19], but also through GrB activity [20–22]. However, granzyme B has also been involved in pathogenesis [23–26]. IL-10 was associated with a lack of parasite control but may also play a role in the control of excessive inflammatory response [27–30]. Despite considerable progress in our understanding of cellular responses involved in protecting against human *Leishmania* infection, the immune profiles associated with protection are still unclear and the immune correlates of protection in human leishmaniasis have yet to be defined.

The objective of this study was to establish and follow a prospective cohort of individuals at risk for ZCL, living in endemic areas of *L*. *major* transmission with varying infection prevalence, evaluate markers of exposure to *Leishmania* infection (LST and scar presence), analyze IFN-γ, IL-10, and GrB levels in *in vitro* PBMC cultures, estimate the incidence of ZCL after the transmission season, and ultimately identify immunological markers associated with resistance or susceptibility to the infection.

## Material and methods

### Ethics statement

All participants provided written informed consent to participate in the study and undergo sample collection and analyses. For individuals under the age of 18, a written informed consent was obtained from a parent or legal guardian. Participants with immunosuppressive diseases other than leishmaniasis, those undergoing long-term treatment, pregnant women as well as children under the age of 5 were excluded from the study due to ethical considerations.

The study protocol was reviewed and approved by the ethical committee of Institut Pasteur de Tunis as well as the Ministry of Health and its regional representatives in Central Tunisia (protocol number 07±0018).

### Study area

The study was carried out in an endemic area for ZCL caused by *L*. *major* zymodeme MON25 [31], situated in the arid zone of central Tunisia, a climatic transition between the Mediterranean zone and the Sahara region, in two governorates: Sidi Bouzid and Kairouan. The study area included five villages: Mbarkia and Dhouibet from Sidi Bouzid, Mnara, Msaadia and Ksour from Kairouan. The selection of these villages was primarily based on the nature of the foci (old versus recent).

The five villages were categorized into different levels of endemicity based on the history of transmission. The old foci, where transmission had lasted for more than 20 years, and the community had become almost immunized with individuals having acquired the infection/lesions. This was the case in Mnara, where the prevalence of lesions was less than 2%, with most cases occurring among young, susceptible children. The new foci, where transmission had begun within a range of less than 5 years to 15 years. This was the case for the remaining villages (Mbarkia, Dhouibet, Ksour, Msaadia) where the entire population was susceptible. All age groups exhibited lesions and the prevalence ranges from around 4% to 20%, based on statistics provided by the national program for the control of cutaneous leishmaniasis at the Primary Health Care Direction, Ministry of Health, Tunisia. In the same study area, using the LST reactivity, Bettaieb et al. [32] demonstrated a significantly higher prevalence of *L*. *major* infection, in the old focus, compared to the recent foci. No differences were observed regarding this parameter between the 4 recent foci [32]. These data emphasize the significance of the previous history of transmission within a specific geographic area.

### Study population and design

The population enrolled in this study included 790 individuals, comprising 359 males and 431 females, aged from 7 to 20 years (mean age 12.5 ± 3.5). They were randomly selected from the endemic study area [32].

At enrollment, during April 2009, physical and detailed skin examinations were performed on each participant. The history of ZCL was assessed based on the presence of typical scar(s), and blood samples were taken before LST administration.

An active case detection of ZCL was carried out between October 2009 and May 2010 (during the ZCL emergence season). Diagnosis of CL was established by dermatologists using clinical criteria and the demonstration of *Leishmania* parasites in Giemsa-stained dermal smears by microscopy.

### Parasite culture

*L*. *major* parasites (MHOM/TN/94/GLC94, zymodeme MON25) were cultured on Novy–Nicolle–McNeal medium at 26°C and progressively adapted to RPMI 1640 medium (Sigma, St Louis, Mo) containing 2mM L-glutamine (Sigma), 100 U/mL penicillin (Sigma), 100mg/mL streptomycin (Sigma) and 10% heat-inactivated fetal calf serum (FCS) (Invitrogen, Cergy Pontoise, France). Metacyclic promastigotes were isolated from day 6 stationary cultures by a discontinuous Ficoll gradient as previously described [33] and used for *in vitro* PBMCs stimulation.

### Leishmanin skin test (LST)

The antigen used in the LST was obtained from the Pasteur Institute of Iran prepared from Iranian *L*. *major* strains. LST was performed by intradermal injection in the inner surface of the forearm of 0.1 mL of leishmanin (suspension containing 5 x 10^6^ *L*. *major* promastigotes/ml in 0.5% phenol saline). Readings were taken 48 to 72 hours later using the ballpoint-pen technique of Sokal to determine the 2 diameters of the induration [34]. The LST result was considered positive if the mean of the two measurements was five mm or more [35].

### Isolation of human peripheral blood mononuclear cells and cell stimulation

PBMCs were separated from heparinized blood samples using Ficoll/Hypaque (GE Healthcare, Uppsala) density gradient centrifugation. 1.10^6^ Cells/mL were stimulated by live metacyclic *L*. *major* parasite (5 parasites / cell) or PHA (10 μg/mL) as a positive control. Culture supernatants were collected after 48h (for IL-10 detection) or 5 days (for IFN-γ and GrB detection) and conserved at -80°C, until use.

### GrB, IL-10 and IFN-γ detection in culture supernatants

GrB was measured in culture supernatants using commercially available human Granzyme B ELISA set (MABTECH AB, Sweden) according to manufacturer’s instructions. For IL-10 and IFN-γ detection, OptEIA ELISA Sets (BD Biosciences Pharmingen) were used. The results were interpolated from a standard curve using recombinant cytokines and expressed in pg/mL.

### Statistical analysis

We used the shapiro-wilk test to check for normality distribution of continuous variables. Comparisons between groups were based on the median and the nonparametric equality-of-medians test was applied. We tested the existence of a significant difference between proportions using either the chi-square or Fisher’s exact tests. p values ≤0.05 were considered statistically significant. The Mann-Whitney test was used to test for significant differences between groups (i.e., differences between stimulated and non-stimulated cultures). Statistical analyses were undertaken using STATA/IC 11.0 (StataCorp, College Station, TX) or GraphPad Prism.

## Results

### 1. Clinical and immunological features in individuals residing in ZCL endemic areas prior to the parasite transmission season

#### 1.1. Prevalence of LST positivity and scar presence at baseline

A Total of 790 individuals were enrolled from 5 foci showing different prevalence rates of *L*. *major* infection. They underwent a physical examination to detect typical scars and were subjected to a skin test.

The clinical examination conducted at the onset of our study indicated that 24% of individuals in our cohort had a previous history of ZCL, as evidenced by the presence of typical scars (Table 1). The latter is usually characterized by an atrophic, hypo-pigmented catching the eye with irregular borders scar in the uncovered parts of the body. Other variants could be hyper-pigmented or keloid leading to stigma particularly for females and facial locations.

**Table 1:** Clinical and immunological parameters of *L*. *major infection* in individuals living in endemic areas.

	Study Cohort n = 790	Old Focus		Recent Foci					
		Mnara n = 184		Mbarkia n = 100		Dhouibet n = 159		Ksour n = 225	Msaadia n = 122
Scar+ n = 190	190 (24%)	74 (40%)		13 (13%)		33 (21%)		52 (23%)	18 (15%)
P value		p < 0.001							
LST1^+^ n = 431	431 (54%)	181 (98%)		38 (38%)		66 (42%)		98 (44%)	48 (39%)
P value		p < 0.001							
LST1+ Scar+ n = 163	163 (21%)	72 (39%)	11 (11%)		25 (16%)		39 (17%)		16 (13%)
P value		p < 0.001							
LST1+ Scar- n = 268	268 (34%)	109 (59%)	27 (27%)		41 (26%)		59 (26%)		32 (26%)
P value		p < 0.001							
LST1- Scar+ n = 27	27 (3%)	2 (1%)	2 (2%)		8 (5%)		13 (6%)		2 (2%)
P value		p = 0.04							
LST1- Scar- n = 332	332 (42%)	1 (1%)	60 (60%)		85 (53%)		114 (51%)		72 (59%)
P value		p < 0.001							

790 participants living in endemic areas of ZCL were followed up over 1 year throughout one season of *L*. *major* transmission. Parameters such as Leishmanin Skin Test at enrollment, before the transmission season (LST1) and the presence of typical scars (Scar) were monitored at the beginning of the study. Donors were subdivided into 4 clinical groups, according to the LST1 (LST1+/−) and Scar (Scar+/−). Numbers and proportions of each group are shown. Statistical significance was assigned to a value of p<0,05.

The overall prevalence of individuals with a positive LST reaction was 54.5%, regardless of the presence of ZCL scars (Table 1).

Based on the combination of the two parameters LST and scar, we observed the following distribution within the cohort: 21% of cured individuals (LST+/Scar+), 34% had asymptomatic infection (LST+/Scar-) and 42% were naïve (LST-/Scar-) (Table 1). 3% of the cohort exhibited typical scars and tested negative for LST. This could be explained either by the gradual loss of the LST over time or by the non-specific nature of the scar.

The comparison among the 5 foci revealed a significant difference in terms of ZCL scar presence and LST positivity (p<0.001 for both parameters). Indeed, the old focus was significantly different from the other recent foci exhibiting the highest prevalence of ZCL scars and LST positive tests (40% and 98%, respectively) (p<0.001). In the recent foci, only 13 to 23% of individuals had ZCL scars, and 38 to 44% had a positive LST (Table 1). The analysis of both scar and LST results showed higher percentages of cured (39%) and asymptomatic individuals (59%) in the old focus in comparison to the recent ones (11 to 17% and 26%, respectively) (p<0.001). In the old focus, only 1% of individuals were naïve, whereas in recent foci, the percentage ranged from 51 to 60% (Table 1).

These results demonstrate a notable disparity in parasite exposure and the percentage of individuals with a history of ZCL, between old and recent foci with significantly higher rates observed in the old focus. Additionally, asymptomatic infection appears to be more common than symptomatic infection in both old and recent foci (p = 0,008 for old focus; p = 0,04 for recent foci).

#### 1.2. IFN-γ, IL-10 and GrB responses to L. major parasites in individuals residing in ZCL endemic area: Analysis in the entire cohort and by endemic focus

We initially analyzed the immune responses in the total cohort, and subsequently in each endemic ZCL focus. We evaluated the ability of live *L*. *major* metacyclic promastigotes to induce the production of IFN-γ as a Th1 specific cytokine, IL-10 as a Th2 cytokine or as a cytokine associated with regulatory T (Treg) cell responses and GrB, as a marker of cytotoxic activity, by PBMCs from a total of 765 individuals (all five foci).

We opted to stimulate PBMCs with live parasites instead of soluble *Leishmania* antigen (SLA), which is commonly used for PBMC stimulation *in vitro*, mainly to prevent the loss of important parasite antigens. Some antigens in the live parasite or released (excreted/secreted) by the parasite could play a role in the induced immune response and generate a memory T cell response [36–38]. Furthermore, inoculation of humans with live parasites (known as leishmanization) is the only method that effectively induces protection against the disease, as opposed to the ineffective vaccination with killed parasite antigens [39]. We have already demonstrated that *in vitro* stimulation of PBMC from individuals immune to CL by live parasites is associated with a significant *Leishmania*-specific IFN-γ response [16].

**Fig 1:**
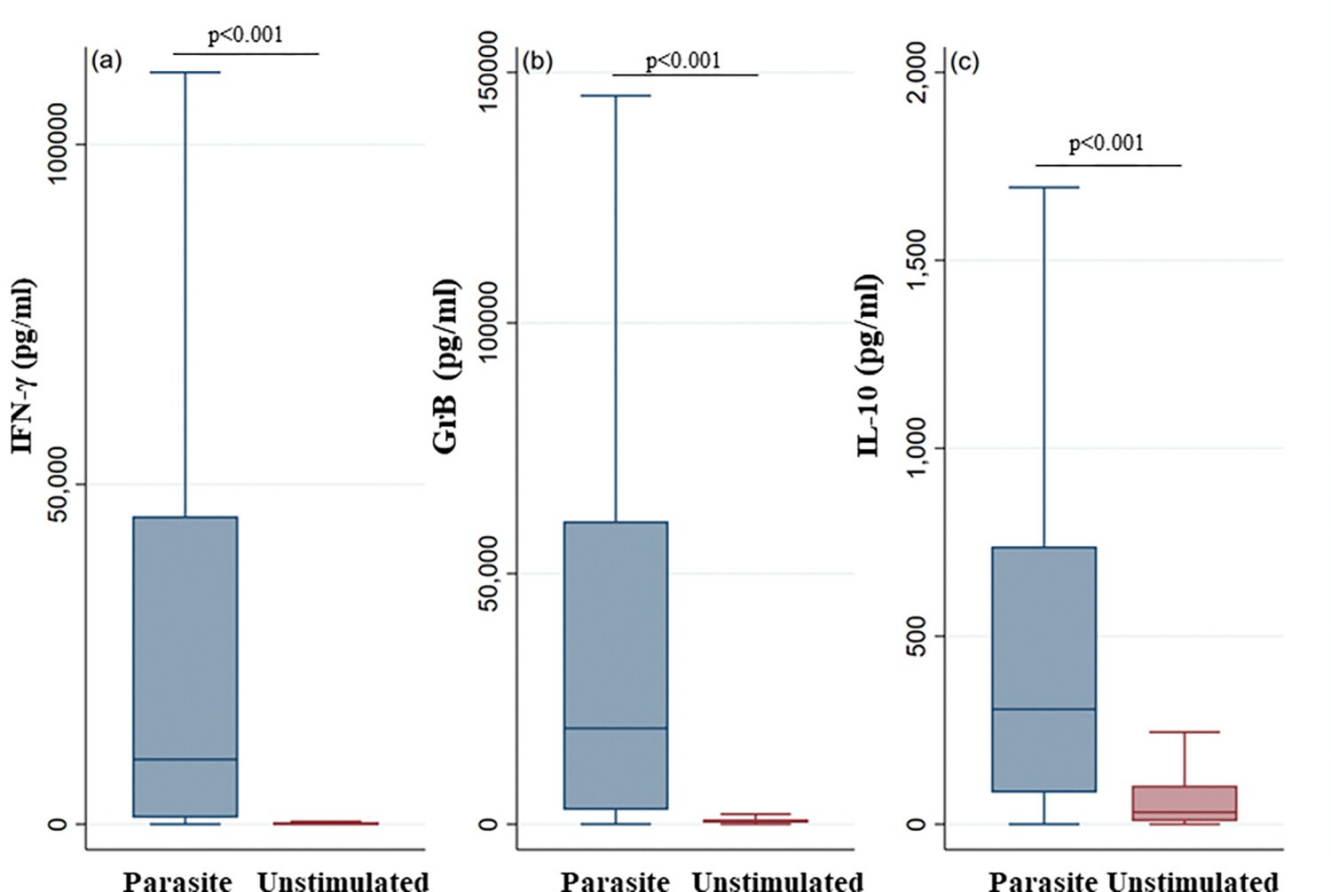
IFN-γ, GrB and IL-10 production in response to parasite stimulation in individuals living in areas endemic for *L*. *major* infection (entire cohort). IFN-γ (a), GrB (b) and IL-10 (c) were quantified by ELISA in the culture supernatants of PBMCs from donors residing in areas endemic for *L*. *major* infection. The supernatants were collected after 48h (for IL-10 detection) or 5 days (for IFN-γ and GrB detection) following stimulation with *L*. *major* parasites (5 parasites/cell). The box represents the 25^th^ and 75^th^ percentiles. The median is represented by a solid horizontal line. The whiskers of the box show the 1^st^ percentile to the 99^th^ percentile. Statistically significant differences between the median of stimulated and non-stimulated cultures were assessed by the non-parametric median test. Results of the median test are shown. Statistical significance was assigned to a value of p<0,05.

Specific and significant IFN-γ and GrB responses, were detected in the culture supernatants of PBMCs from the study population (n = 705) after parasite stimulation (IFN-γ: median [IQR] in stimulated versus unstimulated cultures: 9533 pg/mL [961–45322]; 35 pg/mL [2–175]. GrB: 19138 pg/mL [2858–60451]; 504 pg/mL [305–1001]) (p<0.001) (Fig 1).

Interestingly, a strong correlation between IFN-γ and GrB was observed (Spearman’s correlation coefficient, rho = 0.8). IL-10 was also assessed in the PBMC culture supernatants after stimulation, and significantly higher levels were detected in stimulated cultures (306pg/mL [84–739]) compared to unstimulated cultures (32 pg/mL [8–103]) (p<0.001) (Fig 1).

**Fig 2:**
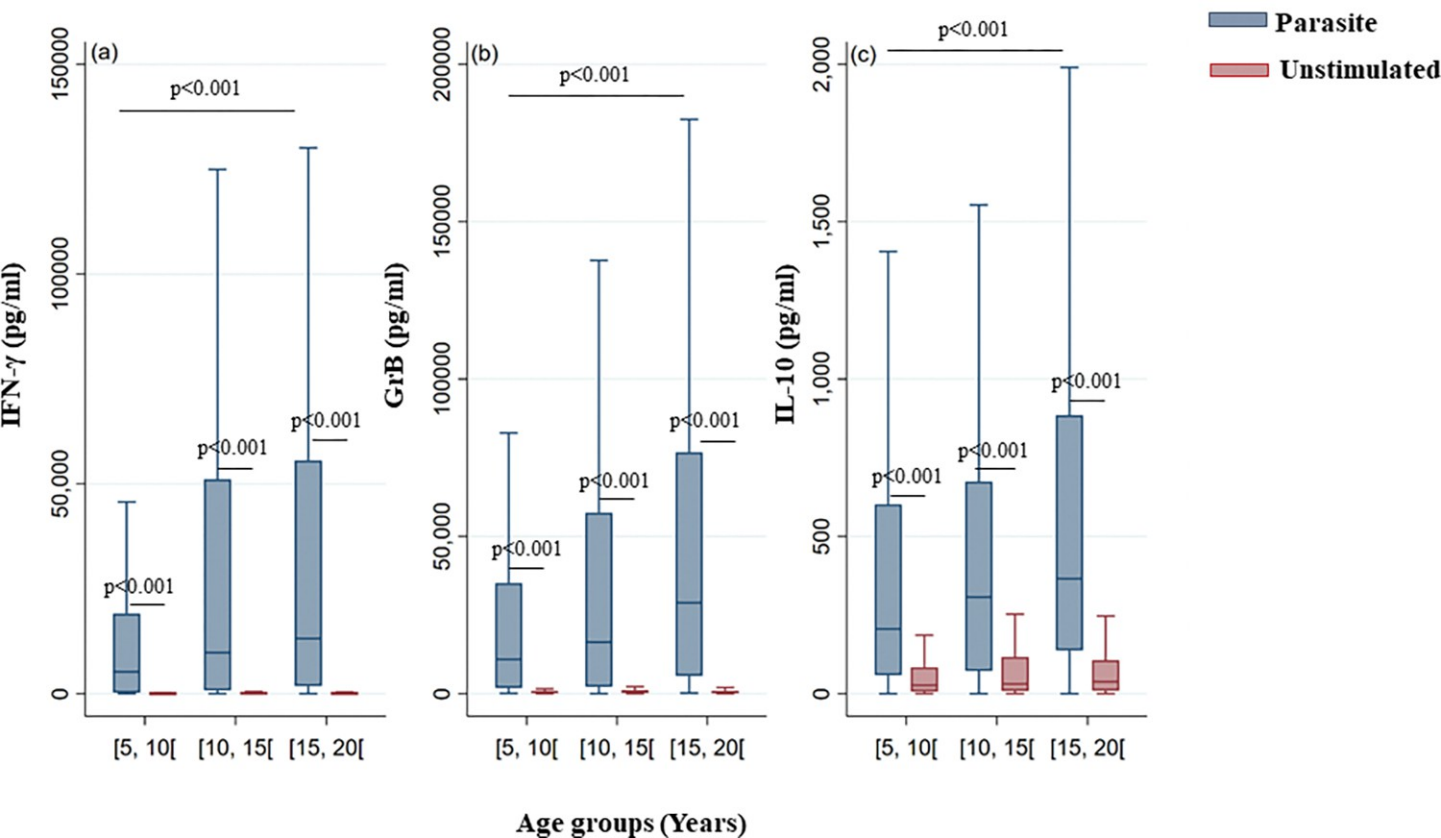
IFN-γ, GrB and IL-10 production in response to parasite stimulation based on age groups of the study population. IFN-γ (a), GrB (b) and IL-10 (c) were quantified by ELISA in the culture supernatants of PBMCs from donors residing in areas endemic for *L*. *major* infection. Donors in the cohort study were divided based on age groups: more than or equal to 5 and less than 10 years ([5–10[), more than or equal to 10 and less than 15 years ([10–15[) and more than or equal to 15 and less than 20 years ([15–20[). The box represents the 25^th^ and 75^th^ percentiles. The median is represented by a solid horizontal line. The whiskers of the box show the 1^st^ percentile to the 99^th^ percentile. Statistically significant differences between the median of stimulated and non-stimulated cultures were assessed by the non-parametric median test. Results of the median test are shown. Statistical significance was assigned to a value of p<0,05.

Our results also demonstrated an age-associated increase in the production of IFN-γ, GrB and IL-10 (p = 0.002, p = 0.001 and p = 0.019, respectively) (Fig 2). This result was specific to parasite stimulation, as no age-associated increase was observed in unstimulated cultures.

**Fig 3:**
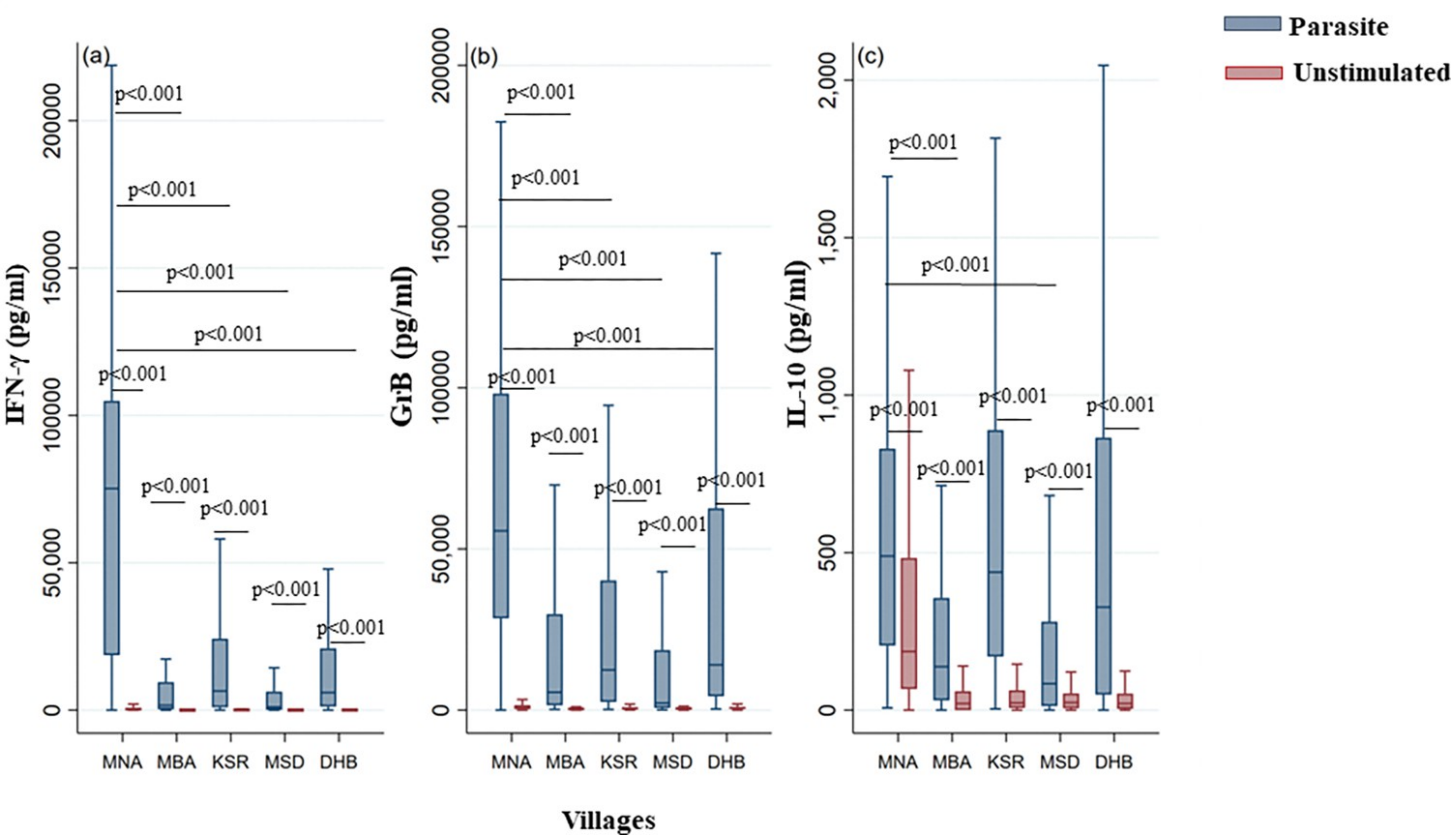
IFN-γ, GrB and IL-10 production in response to parasite stimulation in individuals living in old versus recent ZCL foci. IFN-γ (a), GrB (b) and IL-10 (c) were quantified by ELISA in the culture supernatants of PBMCs from donors residing in an old ZCL focus (MNA) or recent ZCL foci (MBA, KSR, MSD, DHB). The box represents the 25^th^ and 75^th^ percentiles. The median is represented by a solid horizontal line. The whiskers of the box show the 1^st^ percentile to the 99^th^ percentile. Statistically significant differences between the median of stimulated and non-stimulated cultures were assessed by the non-parametric median test. Results of the median test are shown. Statistical significance was assigned to a value of p<0,05.

We next assessed the influence of the past history of transmission (old versus recent foci) on the IFN-γ, IL-10 and GrB responses against the parasite, in our cohort. In addition to the significantly higher levels observed in stimulated cultures compared to unstimulated cultures in each focus, we have demonstrated a significant difference in IFN-γ, IL-10 and GrB responses between the old focus and the recent ones. Indeed, PBMCs from individuals living in the old focus, produced significantly higher IFN-γ, IL-10 and GrB levels when stimulated with live *L*. *major* parasites, compared to the 4 recent foci (p<0.001 for the three markers) (Fig 3). In Msaadia where ZCL cases appeared most recently, we detected the lowest levels of IFN-γ, GrB and IL-10 in culture supernatants (p<0.001). These results indicate that the cytokine and GrB responses are significantly higher in endemic area where exposure to the parasite and the proportion of individuals with a history of ZCL are the highest.

#### 1.3 Association between LST results/Scar presence and cytokine/GrB production

**Fig 4:**
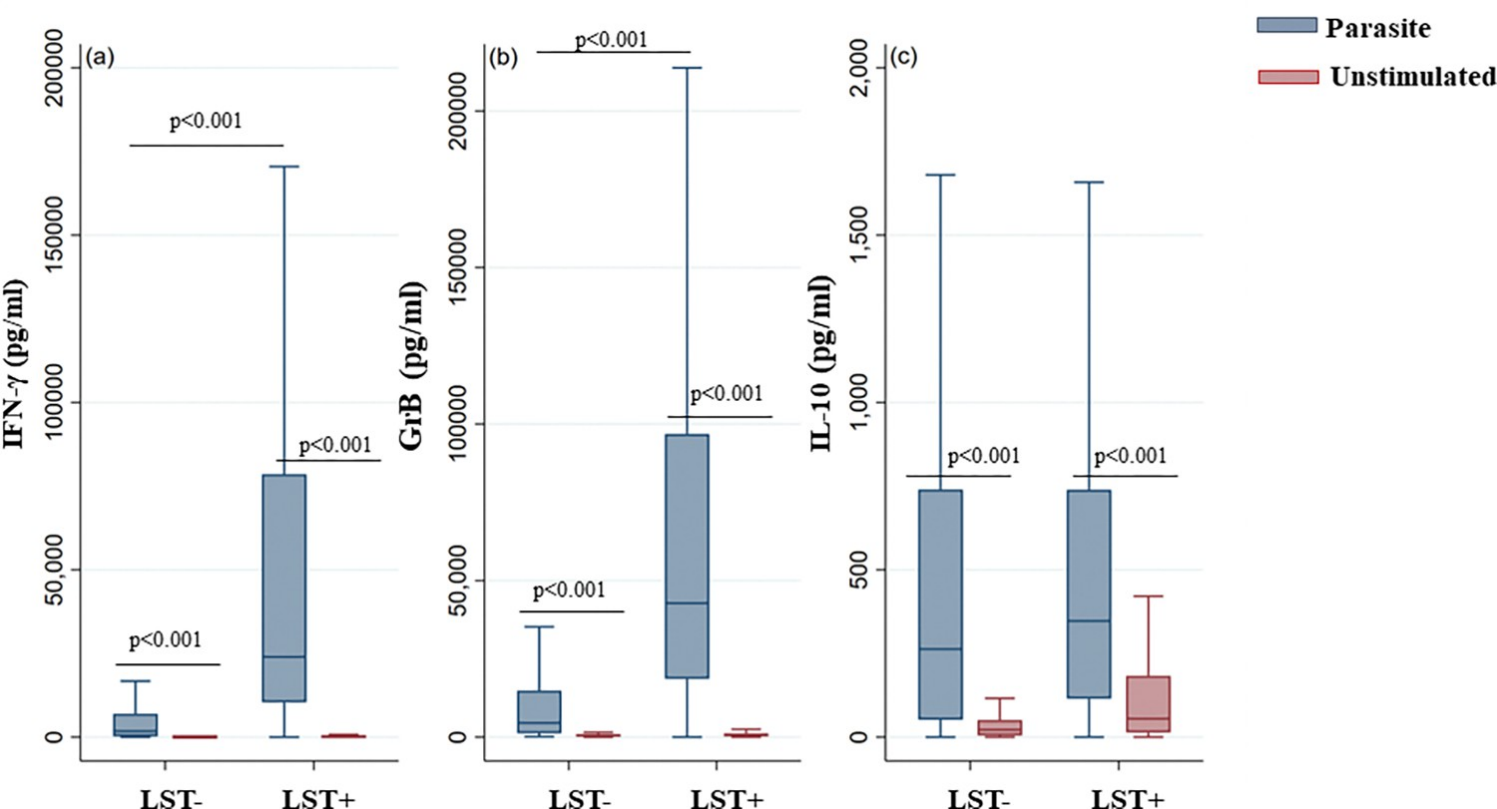
IFN-γ, GrB and IL-10 levels produced in response to parasite stimulation based on LST reactivity (entire cohort). IFN-γ (a), GrB (b) and IL-10 (c) were quantified by ELISA in the culture supernatants of PBMCs from donors residing in areas endemic for *L*. *major* infection. The donors were subdivided based on LST reactivity (LST+ and LST- groups). The box represents the 25^th^ and 75^th^ percentiles. The median is represented by a solid horizontal line. The whiskers of the box show the 1^st^ percentile to the 99^th^ percentile. Statistically significant differences between the median of stimulated and non-stimulated cultures were assessed by the non-parametric median test. Results of the median test are shown. Statistical significance was assigned to a value of p<0,05.

IFN-γ, IL-10 and GrB production by parasite-stimulated PBMCs was analyzed with regards to LST reactivity in the study population (all five villages combined). As expected, our results showed a significant increase in the levels of IFN-γ and GrB produced in response to parasite stimulation among LST+ individuals compared to LST- individuals (p<0.001) (Fig 4). However, although IL-10 levels were higher in LST+ individuals than in LST- individuals, the difference was not statistically significant.

**Fig 5:**
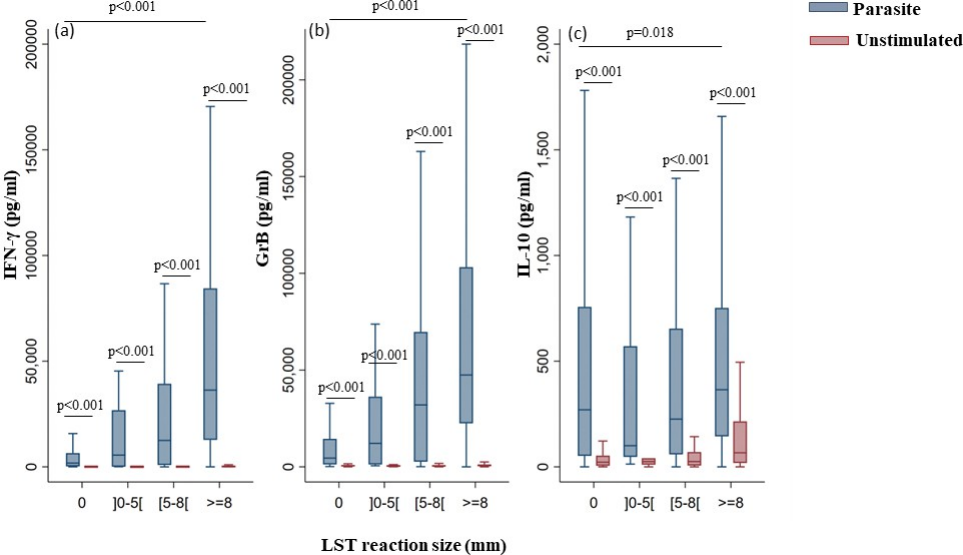
IFN-γ, GrB and IL-10 levels produced in response to parasite stimulation based on LST reaction size. IFN-γ (a), GrB (b) and IL-10 (c) were quantified by ELISA in the culture supernatants of PBMCs from donors residing in areas endemic for *L*. *major* infection. Donors in the cohort study were subdivided based on LST reaction size: 0mm (0), more than 0 and less than 5mm (]0–5[), more than or equal to 5 and less than 8mm ([5–8[) and more than or equal to 8mm (> = 8). Statistically significant differences between groups are shown. The box represents the 25^th^ and 75^th^ percentiles. The median is represented by a solid horizontal line. The whiskers of the box show the 1^st^ percentile to the 99^th^ percentile. Statistically significant differences between the median of stimulated and non-stimulated cultures were assessed by the non-parametric median test. Results of the median test are shown. Statistical significance was assigned to a value of p<0,05.

Interestingly, we observed an increase in IFN-γ and GrB levels according to LST reaction size (p<0.001 for IFN-γ and GrB). Similarly, the subjects with an induration size greater than zero also exhibited a proportional increase in IL-10 levels depending on the LST reaction size (p = 0.018) (Fig 5).

**Fig 6:**
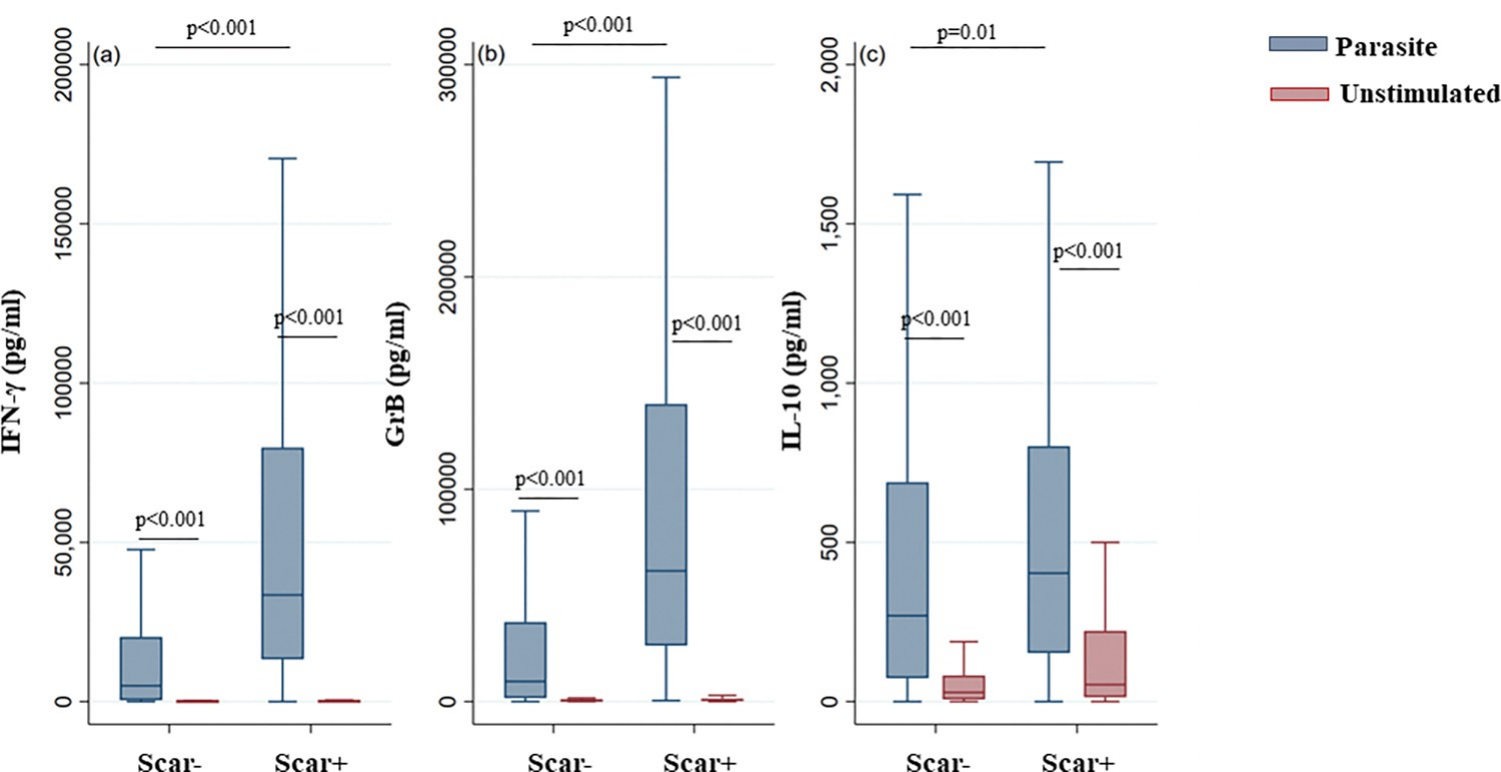
IFN-γ, GrB and IL-10 production in response to *L*. *major* stimulation in individuals with or without ZCL scars. IFN-γ (a), GrB (b) and IL-10 (c) were quantified by ELISA in the culture supernatants of PBMCs from donors residing in areas endemic for *L*. *major* infection. Donors in the cohort study were subdivided based on the presence (Scar-) or absence (Scar+) of ZCL scars. The box represents the 25^th^ and 75^th^ percentiles. The median is represented by a solid horizontal line. The whiskers of the box show the 1^st^ percentile to the 99^th^ percentile. Statistically significant differences between the median of stimulated and non-stimulated cultures were assessed by the non-parametric median test. Results of the median test are shown. Statistical significance was assigned to a value of p<0,05.

In addition, and as expected, IFN-γ, IL-10 and GrB production in response to *L*. *major* stimulation was significantly higher in Scar+ individuals compared to what was observed in Scar- individuals (IFN-γ, GrB: p<0.001; IL-10: p = 0.01) (Fig 6).

**Fig 7:**
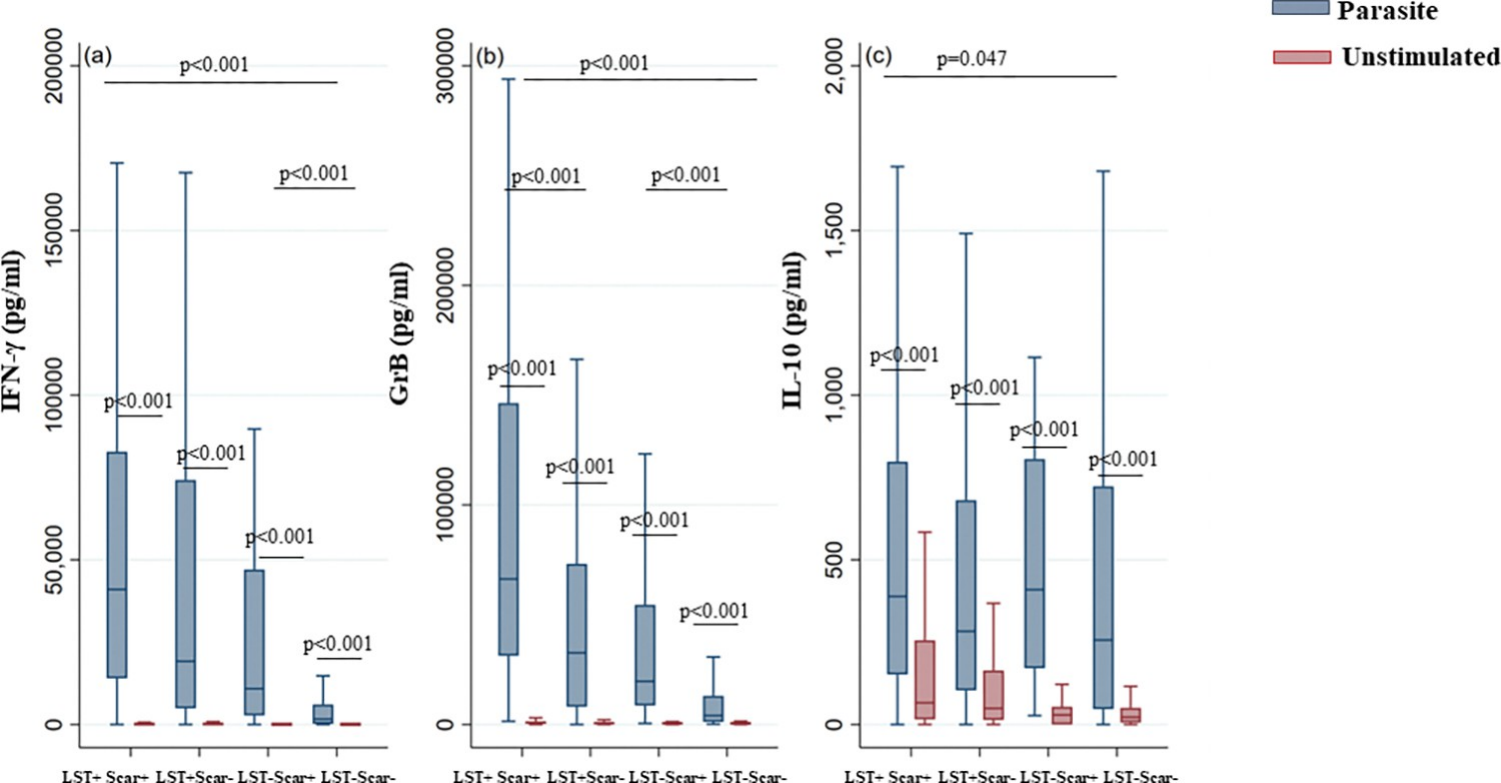
IFN-γ, GrB and IL-10 production in response to *L*. *major* stimulation in LST+/Scar+, LST+/Scar-, LST-/Scar+ and LST-/Scar- individuals. IFN-γ (a), GrB (b) and IL-10 (c) were quantified by ELISA in the culture supernatants of PBMCs from donors residing in areas endemic for *L*. *major* infection. Donors in the cohort study were subdivided based on both scar presence and LST results (LST+/Scar+, LST+/Scar-, LST-/Scar+ and LST-/Scar- groups). The box represents the 25^th^ and 75^th^ percentiles. The median is represented by a solid horizontal line. The whiskers of the box show the 1^st^ percentile to the 99^th^ percentile. Statistically significant differences between the median of stimulated and non-stimulated cultures were assessed by the non-parametric median test. Results of the median test are shown. Statistical significance was assigned to a value of p<0,05.

Finally, the analysis of IFN-γ, IL-10 and GrB production in groups defined by both scar and LST results showed significantly higher levels for IFN-γ and GrB in groups of individuals with immunity to *Leishmania* infection (LST+/Scar+ and LST+/scar-) compared to naïve individuals (LST-/Scar-) (p<0.001) (Fig 7). For IL-10, the difference between the three groups was marginally significant (p = 0.047). Interestingly, the production of GrB was higher in cured subjects compared to asymptomatic ones (p<0.001).

It should be noted that in individuals who remained LST negative despite the presence of scars (LST-/Scar+), we observed significantly higher levels of IFN-γ and GrB compared to naïve individuals (p = 0.001) (Fig 7). This result confirms that scars are ZCL specific despite LST negativity. However, these levels were lower than those observed in LST+ individuals (p<0.001).

These results clearly demonstrated that IFN-γ and GrB are reliable markers of immunity to *L*. *major*. In endemic areas of ZCL, IFN-γ and GrB can differentiate between individuals who have developed immunity to *Leishmania* infection and those who are naïve. However, IFN-γ and GrB were also detected, albeit at lower yet significant levels compared to immune individuals, in the absence of scar and LST reactivity. This result suggests that these apparently naïve individuals living in endemic areas have had a previous contact with the parasite.

#### 1.4. Evaluation of IFN-γ/IL-10 Balance in the study population, based on LST reactivity

Given the significance of maintaining a balance between inflammatory and regulatory cytokines for the development of protective immunity against leishmaniasis, we calculated the IFN-γ/IL-10 and GrB/IL-10 ratios for individuals in our cohort. Various thresholds were established based on their distribution to investigate the potential association between these ratios and the percentage of LST+ individuals (Table 2). In cases where IL-10 was not detected, we used the ELISA detection threshold to obtain this ratio.

**Table 2:** Association between cytokine ratios and LST reactivity.

IFN-γ/IL-10 ratios								
	**<1**	**[1–8[**		**[8–40[**		**[40–180[**		**> = 180**
LST-	70 (21.5%)	110 (33.8%)		78 (24.0%)		34 (10.5%)		33 (10.2%)
LST+	18 (4.5%)	46 (11.5%)		84 (21.0%)		126 (31.5%)		126 (31.5%)
P value	p< 0.001							
GrB/IL-10 ratios								
	**<13**		**[13–65[**		**[65–296[**		**> = 296**	
LST-	139 (42.8%)		89 (27.4%)		49 (15.1%)		48 (14.8%)	
LST+	54 (13.5%)		85 (21.3%)		122 (30.5%)		139 (34.8%)	
P value	p< 0.001							

Values represent the number and percentage of study subjects according to cytokine ratios and LST reactivity.

Statistically significant association between LST and IFN-γ/IL-10 ratios and LST and GrB/IL-10 ratios were assessed by chi-square test. Statistical significance was assigned to a value of p<0,05.

**Fig 8:**
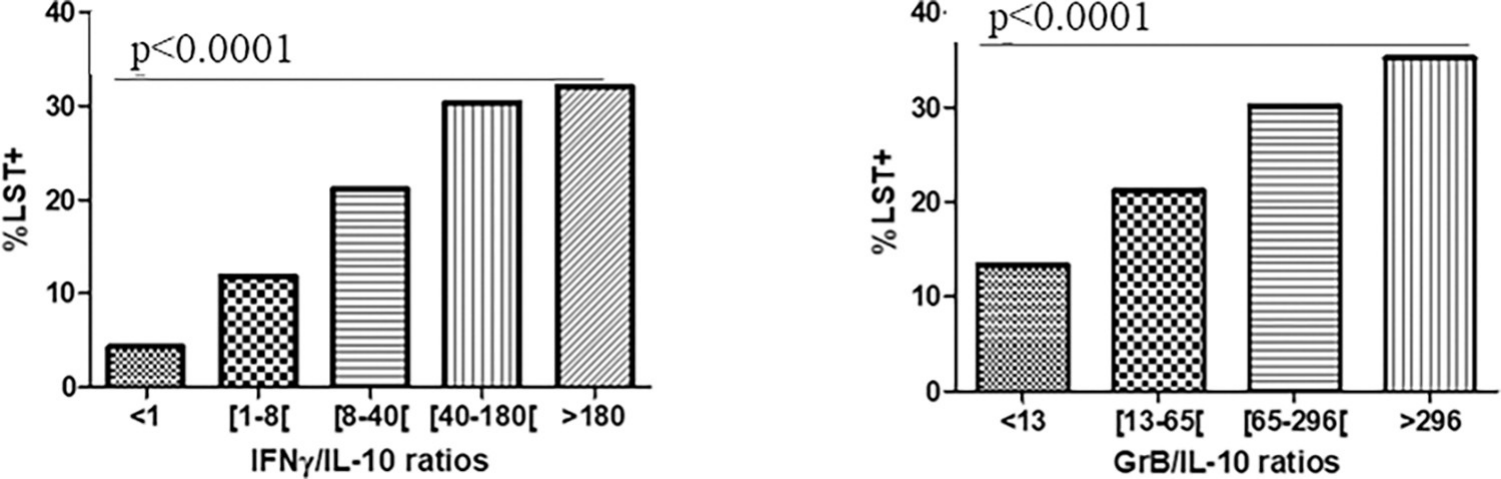
Proportions of LST positive individuals based on IFN-γ or GrB/IL-10 ratio values. Proportions of IFN-γ or GrB/IL-10 ratios are shown in LST+ and LST-subjects. P values were calculated using a non-parametric median test. Statistical significance was assigned to a value of p<0,05.

Our results showed a large heterogeneity among the study subjects, with ratios ranging mainly from 0 to 3443 for IFN-γ/IL-10, and 0,4 to 8057 for GrB/IL-10 ratios. Interestingly, an association was found between the IFN-γ/IL-10 ratios and LST results (p <0.0001). As shown in Table 2, among LST- individuals, the IFN-γ/IL-10 ratios decrease from 21.5% (IFN-γ/IL-10 ratio <1) to 10.2% (IFN-γ/IL-10 ratio > = 180). However, among LST+ individuals, the IFN-γ/IL-10 ratios increase from 4.5% (IFN-γ/IL-10 ratio <1) to 31.5% (IFN-γ/IL-10 ratio > = 180). Furthermore, contrasting results were observed among LST+ and LST- individuals. Indeed, 63% of LST+ individuals exhibited IFN-γ/IL-10 ratios ≥ 40, whereas 79.4% of LST- individuals showed IFN-γ/IL-10 ratios <40 (Table 2). A significant association between GrB/IL-10 and LST reactivity was also shown (p< 0.001) (Table 2). The highest percentages of LST+ individuals were observed for the highest IFN-γ/IL-10 and GrB/IL-10 ratios (Fig 8).

These results allowed us to suggest a clear association between the IFN-γ/IL-10 and GrB/IL-10 balance and the leishmanin skin test reactivity.

### 1.5. Evaluation of IFN-γ and GrB/IL-10 ratios in LST+/Scar+, LST+/Scar-, LST-/Scar+ and LST-/Scar- individuals

**Fig 9:**
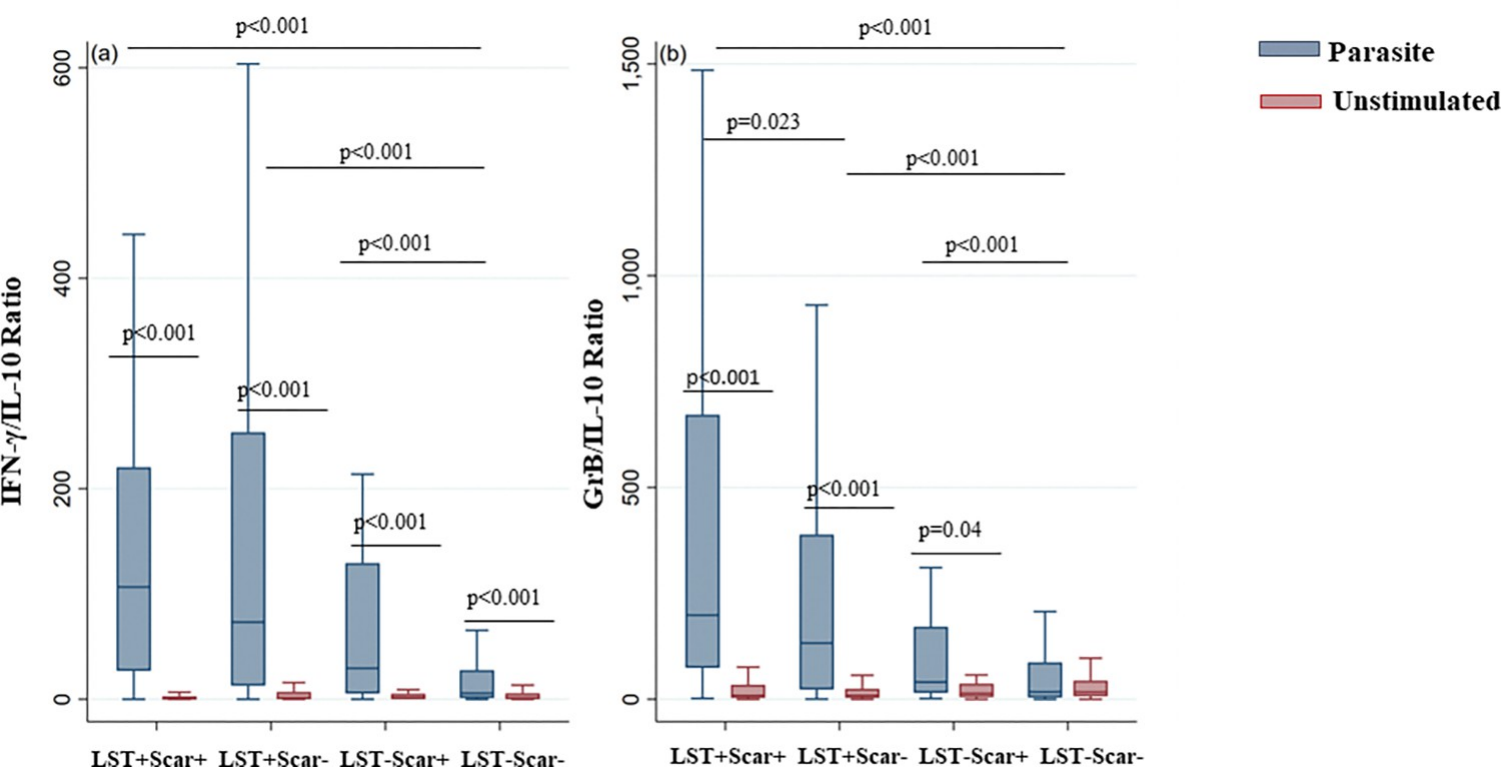
Evaluation of IFN-γ or GrB/IL-10 ratios in LST+/Scar+, LST+/Scar-, LST-/Scar+ and LST-/Scar- individuals. IFN-γ/IL-10 (a) and GrB/IL-10 (b) ratios were calculated using the levels of IL-10, IFN γ and GrB measured by ELISA in culture supernatants of PBMCs from the donors. The ratios’ values are shown for LST+/Scar+, LST+/Scar-, LST-/Scar+ and LST-/Scar- groups. The box represents the 25^th^ and 75^th^ percentiles. The median is represented by a solid horizontal line. The whiskers of the box show the 1^st^ percentile to the 99^th^ percentile. Statistically significant differences between the median of stimulated and non-stimulated cultures were assessed by the non-parametric median test. Results of the median test are shown. Statistical significance was assigned to a value of p<0,05.

We also analyzed the IFN-γ/IL-10 and GrB/IL-10 ratios in LST+/Scar+, LST+/Scar-, LST-/Scar+ and LST-/Scar- individuals and found that these ratios were significantly higher in immune individuals compared to the naïve group (p < 0.001) (Fig 9). Interestingly, the GrB/IL-10 ratio was significantly higher in cured individuals (LST+/Scar+) compared to asymptomatic individuals (LST+/Scar-) (p = 0.023).

### 2. Clinical and immunological features in individuals residing in ZCL endemic areas following the parasite transmission season

#### 2.1. Effect of a transmission cycle on LST conversion

Based on the assumption that LST conversion is an indirect indicator of *L*. *major* infection, a second LST was administered, 1 year after enrollment, to 657 out of the total 790 subjects. Results are shown in Table 3. In the study area (all foci combined), LST conversion (from negative to positive reaction) was observed in 7% (48/657) of the individuals. In the old focus, all 3 individuals who initially tested negative for LST prior to the transmission season showed a conversion to a positive reaction (2%, 3/153), while LST conversions in the recent foci varied between 6 and 14% (Table 3). Furthermore, LST reversion (from a positive to a negative reaction) was observed in 23 individuals (4% of the cohort), all within recent foci.

**Table 3:** LST conversion/reversion following a transmission season in the different ZCL foci.

	Study cohort	Old Focus	Recent Foci			
		Mnara	Mbarkia	Dhouibet	Ksour	Msaadia
LST1(+) / LST2(+)	336 51%	150 98%	30 35%	49 40%	70 37%	37 34%
LST1(-) / LST2(-)	250 38%	0	40 47%	63 52%	90 48%	57 52%
LST1(-) / LST2(+)	48 7%	3 2%	12 14%	8 6%	18 10%	7 6%
LST1(+) / LST2(-)	23 4%	0	3 4%	2 2%	9 5%	9 8%

The distribution of LST conversion/reversion after a transmission season in individuals living in endemic areas is shown (Mnara: old focus; Mbarkia, Dhouibet, Ksour, Msaadia: recent foci). LST1: Leishmanin skin test before the transmission season, LST2: Leishmanin skin test after the transmission season.

#### 2.2. Emergence of new ZCL cases following a transmission season and identification of immune correlates of protection

The cohort was followed up every two weeks to ensure active ZCL case detection. Most reported cases were from recent foci, where the disease occurs with a more diffuse form with a higher number of lesions (Table 4). A total of 29 individuals, which represents 4% of the cohort, developed ZCL (13 were LST1- and 16 were LST1+ with 1 to 9 lesions) (Table 4). Only 2 new cases were recorded in the old focus (both were LST1+ with a lesion diameter of 12,5 and 10 cm). These observations support the view that individuals living in areas endemic for *L*. *major* infection are likely to develop ZCL regardless of their LST status.

**Table 4:** Emergence of ZCL cases following a transmission season in the different ZCL foci.

	Old Focus		Recent Foci							
	Mnara		Mbarkia		Dhouibet		Ksour		Msaadia	
	**LST1+** **(n = 183)**	**LST1- (n = 0)**	**LST1+ (n = 38)**	**LST1- (n = 62)**	**LST1+ (n = 66)**	**LST1- (n = 93)**	**LST1+ (n = 98)**	**LST1- (n = 127)**	**LST1+ (n = 48)**	**LST1- (n = 74)**
**Number of ZCL cases**	2	0	2	3	9	2	2	6	1	2
**Mean number of lesions (SD)**	1 (0)		1.60 (1.34)		4.27 (4.76)		2.75 (2.66)		2.67 (2.08)	

The numbers of new ZCL cases in each focus are represented for both LST1+ and LST1- subjects, and the mean number of lesions, along with the standard deviation, after a transmission season is also shown

To evaluate the correlates of protection against the development of the disease, we compared the levels of IFN-γ, GrB and IL-10 detected at the steady state (prior to the transmission season) between two groups: individuals who developed ZCL after the transmission season (ZCL+) and individuals who did not develop the disease (ZCL-).

**Fig 10:**
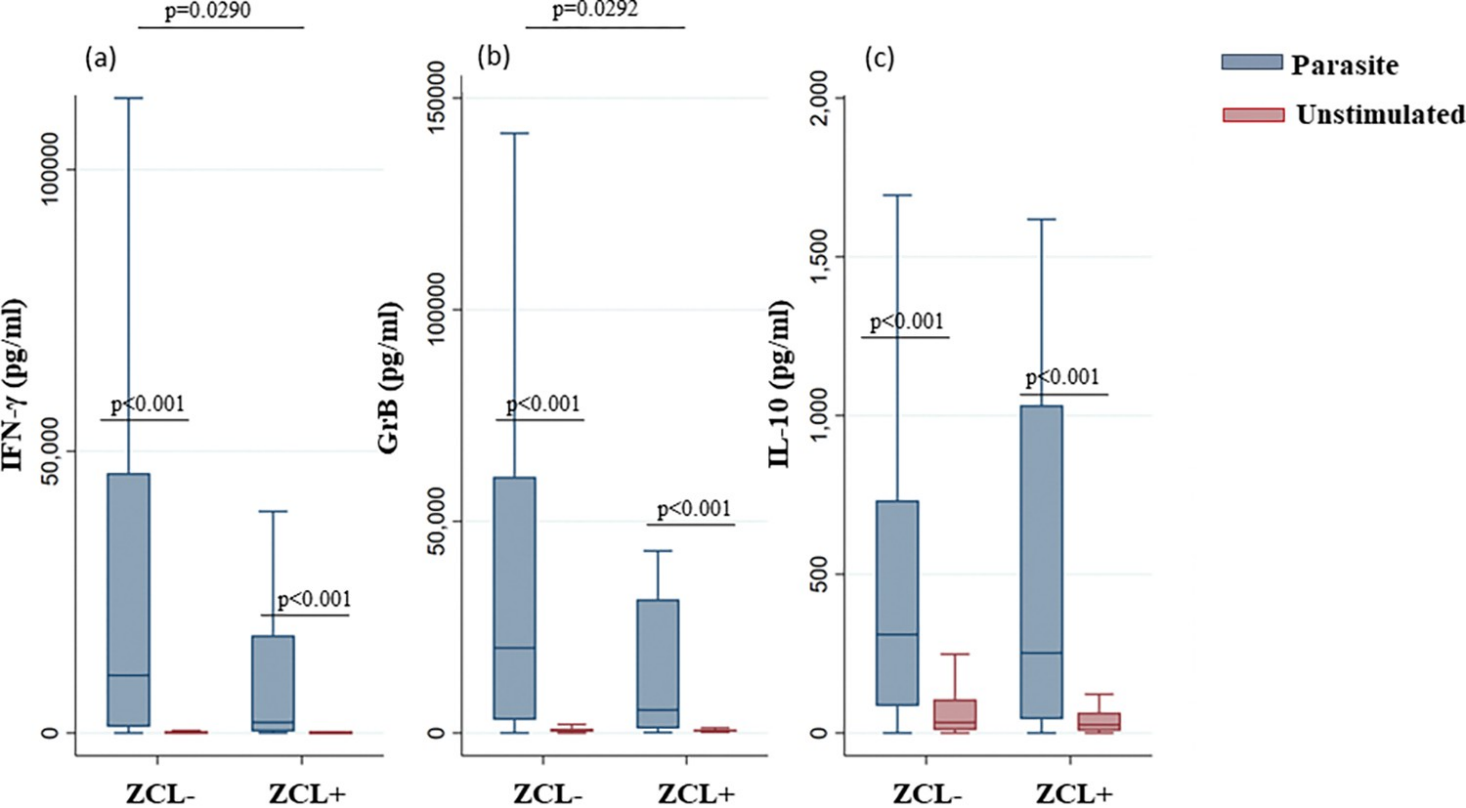
Levels of IFN-γ, GrB and IL-10 in ZCL+ and ZCL- groups within combined foci. IFN-γ (a), GrB (b) and IL-10 (c) were quantified by ELISA, in the culture supernatants of PBMCs from donors residing in areas endemic for *L*. *major* infection. Cytokine and GrB levels were analyzed in individuals who developed ZCL after the transmission season (ZCL+) and those who did not develop the disease (ZCL-). The box represents the 25^th^ and 75^th^ percentiles. The median is represented by a solid horizontal line. The whiskers of the box show the 1^st^ percentile to the 99^th^ percentile. Statistically significant differences between the median of stimulated and non-stimulated cultures were assessed by the non-parametric median test. Results of the median test are shown. Statistical significance was assigned to a value of p<0,05.

Interestingly, we showed a significant increase in IFN-γ levels produced in response to parasite stimulation in the ZCL- group (median [IQR] in stimulated versus unstimulated cultures: 10218 pg/mL [1073–46139]; 35 pg/mL [2–176]) compared to the ZCL+ group (1882.5 pg/mL [253.5–17334.5]; 32.5 pg/mL [3.5–113.5]) (p = 0,0290) (Fig 10). Similar results were shown for GrB levels for the same groups (ZCL-: 20077 pg/mL [3084–60532]; 504 pg/mL [305–1007]. ZCL+: 5436.5 pg/mL [1049.5–31586.5]; 550.5 pg/mL [307–836.5]) (p = 0,0292) (Fig 10). However, there was no significant difference in IL-10 levels between the two groups.

These results suggest that elevated levels of IFN-γ and GrB detected at the steady state could be considered as immunological markers of protection.

### 2.3. IFN-γ/IL-10 ratios and protection

Based on the importance of appropriate levels of IFN-γ and IL-10 in determining the outcome of *Leishmania* infection, we compared the IFN-γ/IL-10 ratios among the subjects who developed the disease.

**Fig 11:**
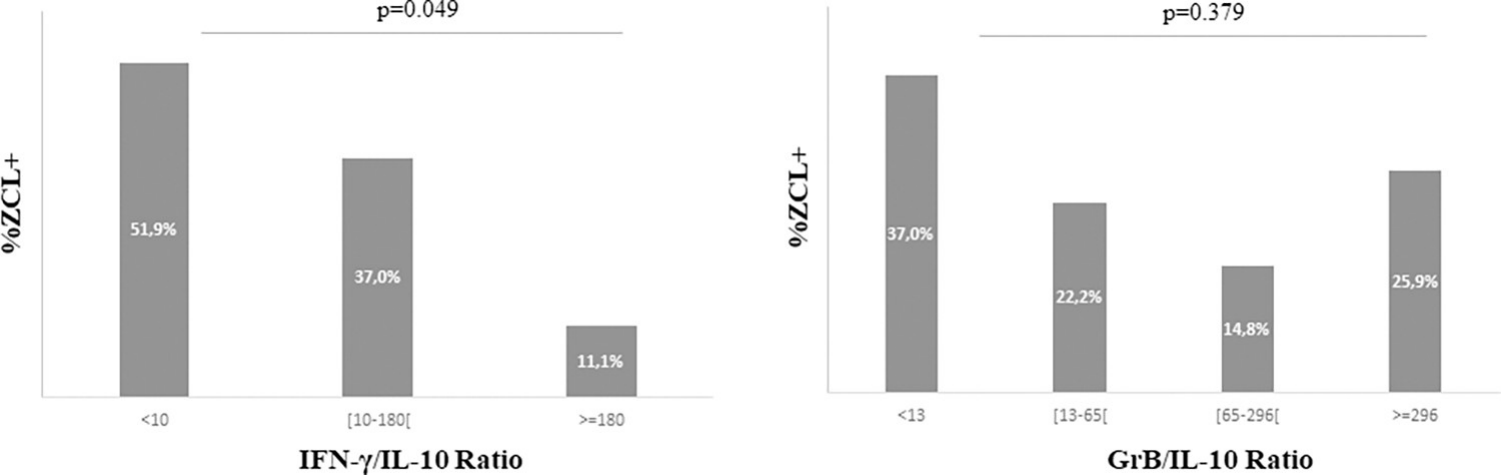
Proportions of ZCL cases based on IFN-γ/IL-10 ratio values. IFN-γ/IL-10 ratios were calculated using the levels of IL-10 and IFN γ measured by ELISA in culture supernatants of PBMCs from ZCL+ donors. Percentages of ZCL+ individuals are shown within each IFN-γ/IL-10 ratio interval. Statistical significance was assigned to a value of p<0,05.

As illustrated in Fig 11, 57.1% of individuals who developed ZCL lesions after a transmission season exhibited the lowest ratios (<10), whereas only 7% of the emerging cases showed very high IFN-γ/IL-10 ratios (≥180). Higher percentages of ZCL- were observed among individuals with the highest IFN-γ/IL-10 (≥180) compared to ZCL+ individuals (Table 5).

**Table 5:** IFN-γ/IL-10 ratios and ZCL development.

	IFN-γ/IL-10 ratios				
	<1	[1–8[	[8–40[	[40–180[	> = 180
**ZCL-**	82 (11.7%)	149 (21.3%)	157 (22.5%)	154 (22.1%)	156 (22.3%)
**ZCL+**	6 (22.2%)	7 (25.9%)	5 (18.5%)	6 (22.2%)	3 (11.1%)

Values in each cell represent the number, and the percentage (in brackets), for each ratio according to ZCL cases.

A significant decrease in the percentage of ZCL cases was observed as the IFN-γ/IL-10 ratios increased (p = 0.049), suggesting an association between this ratio and the protection against the development of the disease (Fig 11). Despite the positive correlation between the baseline IFN-γ and GrB levels (before the transmission season) that we reported above, we found no association between the GrB/IL-10 ratios and ZCL development.

## Discussion

The immunological basis of resistance to infection with *L*. *major* parasites in humans is still not well defined, and the lack of clear correlates of protection remains one of the greatest hurdles to parasite vaccine development. In this study, we investigated the immune correlates of protection against ZCL by conducting a prospective study on a cohort of individuals living in endemic areas for *L*. *major* infection. We analyzed 5 areas with varying endemicity levels: one representing an old focus with high transmission pressure, and the others representing areas with more recent introduction of the parasite. The individuals participating in our study are part of a larger cohort that has been analyzed in other studies conducted in the same foci [5, 32]. Based on LST reactivity, it has been shown that the prevalence of *L*. *major* infection was significantly higher in the old focus compared to the recent ones [5, 32]. There are few studies describing well-defined foci exhibiting different levels of leishmaniasis prevalence [6, 40].

We showed a significantly higher prevalence of ZCL scars and LST positivity, as well as a significantly higher percentage of cured and asymptomatic subjects in the old focus compared to the recent ones. These results were expected, given that the duration of exposure to infecting sand fly bites is much higher in the old focus than in the recent ones. We have also demonstrated that asymptomatic infection is more prevalent than symptomatic infection in both foci. The fact that most individuals bitten by infected sandflies in endemic areas will remain asymptomatic has been reported by others [5, 6, 41]. However, unlike our results which showed a higher percentage of asymptomatic subjects in the old focus, it was suggested that in the context of a low exposure level to parasites, the infection is more likely to be asymptomatic [6]. These discrepancies could be attributed to the evolution of lesions to normal skin over time in the old foci and the challenges of comparing epidemiologic studies conducted in different endemic areas. Such difficulties arise due to differences in transmission rates and the characteristics of the study populations. However, it should be noted that it is not always possible to be certain that LST+ and scar- individuals are asymptomatic. The possibility of missing a scar in an unusual location is theoretically conceivable, as is a scar that has reverted into normal skin. However, given that our study is prospective, with frequent follow-up (every 2 to 4 weeks) for the cohort, and the fact that most of the lesions are in uncovered parts of the body, this probability is low, though not entirely excluded.

When analyzing the total cohort, we observed that while most of individuals with typical scars were LST positive, some of them were LST negative, suggesting that there was not a perfect association between LST reactivity and scars. This observation aligns with findings from a previous epidemiological study, and can be explained by either the reversion of an LST positive reaction over time or non-specific scars [6].

We collected immunological data including LST results and the ability of the PBMCs to produce IFN-γ, GrB, and IL-10 in response to *L*. *major*, prior to the transmission season, in both old and recent foci. We showed that IFN-γ, GrB, and IL-10 responses were significantly higher in the old focus compared to the recent ones. This indicates that subjects living in an old focus who were continuously exposed to infected sand fly bites, were able to display stronger cellular immune responses corresponding to memory T cells that are maintained through the continued presence of parasites [42]. Association between exposure time to *Leishmania* and the IFN-γ response was reported [43].

We found that IFN-γ, GrB, and IL-10 production in response to parasite stimulation increased with age. This result could be explained by older people having a longer time of exposure to infective sand fly bites compared to younger individuals. The effect of age on the immune response against *Leishmania* infection has already been reported [44, 45].

Similarly to our results, in endemic areas for *L*. *major*, the percentage of individuals with high levels of GrB was greater in the group over 6 years old than in those under 6 years old [22]. However, it has been shown that elderly subjects with American tegumentary leishmaniasis produced less IFN-γ and more IL-10 than young patients, probably due to impaired protective immune responses in the elderly [44]. However, we cannot compare the results of this study to ours since different *Leishmania* species are studied and our study population consists only of young subjects.

We showed that both IFN-γ and GrB levels were significantly higher in individuals who had previous contact with the parasite (LST+ individuals, with or without scars). IFN-γ is a crucial cytokine in the control of *Leishmania* infection, and its production has already been reported in LST+ individuals living in an endemic area for *L*. *major* or *L*. *infantum*, as well as *L*. *braziliensis* infection [13, 46–50]. In this work, we did not analyze the cellular sources of IFN-γ. However, in patients with CL the majority of cells expressing IFN-γ were CD4+ and CD8+ T cells [17, 19, 21, 26, 51], while NK cells were the main source of IFN-γ in subjects exposed to *L*. *braziliensis* who did not develop CL [52].

GrB, is a proapoptotic protease that has been associated with both tissue damage and good prognosis in CL and VL patients [20, 22, 23, 53, 54]. It has been described as a good marker of immunity to *L*. *major* infection, as well as a good correlate of protection against intermediate and severe forms of CL due to *L*. *major* [22]. Recently, the detection of GrB was reported in ZCL lesions [53]. Its expression was found to be negatively correlated with the lesion’s age, and its implication in the parasite control was suggested [53]. However, in patients with CL due to *L*. *braziliensis*, a strong positive correlation was found between GrB and lesion size and it has been suggested that this molecule may contribute to an enhanced inflammatory response inducing lesion development [23, 48, 55]. CD4+ T cells producing GrB in response to *L*. *major* excreted/secreted proteins, were reported in healed CL individuals [36, 54]. The simultaneous expression of GrB and IFN-γ by CD8+ T cells was also described in cured patients [56].

Interestingly, we found a positive correlation between IFN-γ and GrB. We previously reported a strong correlation between IFN-γ and GrB in response to *Leishmania* antigens in individuals who have healed from ZCL [54]. Additionally, a positive correlation between IFN-γ and GrB was also reported within ZCL lesions [53].

Furthermore, we observed an increase in IFN-γ and GrB levels according to LST reaction size, suggesting that they can be used as indicators of previous contact with the parasite. These results are in agreement with the positive associations between IFN-γ and GrB levels and LST positivity that we observed in this work. Positive associations between IFN-γ or GrB levels and LST positivity were previously reported by our group and by others [13, 22, 50, 54].

However, it should be noted that some discrepancies were observed between LST and scars results and IFN-γ or GrB responses since the latter were also detected in naïve individuals (LST-/Scar-). Discordance between LST and *in vitro* IFN-γ production was also reported in LST- individuals living in an endemic region for American tegumentary leishmaniasis due to *L*. *braziliensis* [50] and in individuals with subclinical *L*. *chagasi* infection [47]. Boussoffara et al. have shown that variable levels of GrB, sometimes high, were detected in LST- individuals [22].

These results suggest that even in the absence of LST reactivity, both IFN-γ and GrB responses may serve as indicators of previous contact and could be associated with protection. However, we cannot exclude the possibility that the discordance between LST results and *in vitro* tests could be attributed to the presence of suppressor factors, which might explain the absence of the *in vivo* response.

We also evaluated IL-10; a cytokine that has been involved in both susceptibility to leishmaniasis and the regulation of CL development. Several cells produce IL-10, such as activated Th2 cells, monocytes and regulatory T cells (Tr1) [57, 58]. This cytokine is characterized by its suppressor effects on pro-inflammatory cytokine production and T cell responses, and is involved in the parasite persistence in mice [58, 59]. IL-10 is also an important regulatory cytokine in the immune response to human leishmaniasis [15, 60, 61].

Although IL-10 was significantly detected in LST+ individuals, no association was noted between the IL-10 response and LST results, as this cytokine was similarly detected in LST negative individuals. Higher levels of this cytokine were observed in cured and asymptomatic individual compared to naïve subjects, but the difference between the three groups was only marginally significant.

Unlike IL-10 responses, IFN-γ and GrB levels were found significantly higher in cured individuals compared to asymptomatic subjects. Other authors did not find any difference in the intensity of the specific immune response to *L*. *major* between patients who developed lesions and those who had asymptomatic infection [13]. The discrepancy between our work and the latter could be attributed to the different antigen preparations used, namely live parasites versus soluble *Leishmania* antigen, respectively. However, our results were comparable with other studies showing lower IFN-γ responses in asymptomatic individuals compared to individuals healed from CL due to *L*. *braziliensis* [43, 52, 62–64]. Bittar and colleagues observed higher IL-10 levels, as well as lower IFN-γ/IL-10 ratios, in asymptomatic subjects compared to symptomatic individuals [62]. These findings led the authors to suggest that IL-10 in asymptomatic individuals could counter-regulate the effect of IFN-γ and may explain the absence of symptomatic disease. In our study, we also found that IFN-γ/IL-10 ratios were lower in asymptomatic subjects compared to cured individuals.

On the other hand, the evaluation of the balance between IFN-γ and IL-10 showed that the IFN-γ/IL-10 ratios were higher in LST+ individuals compared to the LST- group. Furthermore, low ratios (<1), indicating higher IL-10 than IFN-γ levels, were found in only 4% of LST+ individuals. These results suggest that individuals who developed immunity to *L*. *major* parasites exhibit high IFN-γ/IL-10 ratios, which are favorable indicators for a good prognosis. These results corroborate those described by Carvalho and colleagues, who showed that subjects aged over 60 years, compared to younger subjects (21–30 years old), exhibit a decrease in IFN-γ production and an increase in IL-10. These factors are associated with the spread of leishmaniasis or the development of mucocutaneous form [44]. However, it should be noted that high IFN-γ/IL-10 ratios were also associated with the severity of mucosal leishmaniasis [65].

We also demonstrated that the highest GrB/IL-10 ratios were found in the highest percentage of LST+ individuals, and these ratios were higher in immune individuals compared to naïve subjects. These results are in concordance with the positive correlation found between IFN-γ and GrB responses.

A clinical examination conducted after a season of parasite transmission allowed us to identify new ZCL cases and analyze the impact of the immune status before the transmission season on the development of the disease.

After a transmission season, we recorded 29 new cases of ZCL, accounting for only 4% (29/790) of the cohort. Notably, only two individuals developed the disease in the oldest focus where almost all individuals were likely naturally immunized with infective sandfly bites. Furthermore, within our cohort 23 individuals from recent foci lost reactivity to leishmanin, indicating that this reactivity is not definitive and may diminish over time. This observation has already been documented in the literature [5, 6, 66]. Bettaieb et al. [5] also showed that the rate of LST reversion was significantly higher in recent foci compared to the old focus. The decline of LST reactivity in the recent foci can be attributed to the relatively short history of exposure to infected sandfly bites, while in the old focus, where individuals experience high transmission pressure and continued parasite exposure, LST reversion is not detected.

On the other hand, we have shown that for 47 individuals, the LST became positive, without developing the disease. This is most likely related to an asymptomatic infection. Indeed, as discussed in the work of Ben Salah et al. [6], the incidence of asymptomatic infection can range from 8% to 12% in endemic areas in southern Tunisia.

We observed that 10 individuals did not convert their LST, even though they developed ZCL lesions after the transmission season. This observation confirms what we had already established before the transmission season, as we have shown that 3% of the study cohort had a negative LST reaction in the presence of scars. Such results have already been described in the study by Sadeghian et al. [67], wherein patients with a confirmed diagnosis of CL remained negative for the LST even up to one year after treatment. These findings raise doubts about the reliability of the LST in reflecting potential contact with the parasite. However, it should be noted that protection could still be conferred even when LST reactivity is low (< 5mm), questioning the threshold of LST positivity [6].

It is also interesting to note that among individuals who developed the disease, 13 maintained a positive LST. Furthermore, similar percentages of individuals with a positive LST were found for both groups (ZCL+/ZCL-). However, individuals who developed ZCL showed lower induration diameters compared to those who did not develop the disease, though this difference was not statistically significant. These findings suggest that there might not be a direct link between LST results and protection against the disease. Therefore, LST, when taken alone, may not be a reliable correlate of protection. It is important to note that the interpretation of our results could be influenced by the small number of new cases diagnosed during the follow-up period. These results contradict previous studies, including our work in the Tataouine area, where we demonstrated that a positive LST provides protection against the development of ZCL. Furthermore, we found that this protection increases with the diameter of induration [6].

Nonetheless, other studies have reported that the association between LST and protection was not consistently found [68, 69]. Furthermore, it has been observed that the reactivity to leishmanin, especially when "artificially" induced in vaccination trials in humans, does not correlate with protection [70].

Finally, we have demonstrated that individuals who did not develop ZCL had higher levels of IFN-γ and GrB compared to ZCL+ individuals. These findings suggest that elevated levels of IFN-γ and GrB during the steady state could serve as reliable immunological markers indicating protection. Muniz and colleagues [52] previously reported that protection against the development of CL was associated with IFN-γ production rather than with LST positivity. Additionally, our study revealed that more than half of the ZCL+ subjects exhibited low IFN-γ/IL-10 ratios, while only 7% of them showed high ratios, indicating a noteworthy association between IFN-γ/IL-10 ratios and protection.

In conclusion, this study represents the first comprehensive analysis of IFN-γ, GrB, and IL-10 in a cohort study within well-defined endemic regions for *L*. *major* infection, considering variations in the level of transmission pressure. This research sheds light once again on the complexities of immunity to *L*. *major* parasites and offers valuable insights into the immunological responses associated with prior contact with the parasite and potential indicators of protection against the disease. The findings underscore the potential of IFN-γ and GrB levels, along with IFN-γ/IL-10 ratios, as indicators of protection. Moreover, the correlation between increased IFN-γ and GrB levels and the size of the LST reaction suggests their utility in identifying previous exposure to the parasite. Our results confirm that relying solely on LST may not be sufficient as an indicator of previous contact with the parasite or as a protective correlate, especially in areas where the history of exposure to infected sandfly bites is short. Instead, integrating IFN-γ and/or GrB responses with LST results could enhance the assessment of the immune response to a vaccine in human studies.
